# The learning curve to ROSA: cases needed to match the surgery time between a robotic-assisted and a manual primary total knee arthroplasty

**DOI:** 10.1007/s00590-023-03554-6

**Published:** 2023-04-27

**Authors:** Eustathios Kenanidis, Panagiotis Boutos, Olga Sitsiani, Eleftherios Tsiridis

**Affiliations:** 1 Academic Orthopaedic Department, Aristotle University Medical School, General Hospital Papageorgiou, Ring Road Efkarpia, 56403 Thessaloniki, Greece; 2 Centre of Orthopaedic and Regenerative Medicine (CORE), Center for Interdisciplinary Research and Innovation (CIRI)-Aristotle University of Thessaloniki (AUTH), Balkan Center, Buildings A & B, Thessaloniki, 10th km Thessaloniki-Thermi Rd, P.O. Box 8318, 57001 Thessaloniki, Greece; 3 Tsiridis Orthopaedic Institute-ICAROS clinic, Thessaloniki, Greece; 4 Thessaloniki, Greece

**Keywords:** ROSA, Robotic TKA, Robotic knee arthroplasty, TKA, Total knee arthroplasty, Surgery time, Learning curve

## Abstract

**Purpose:**

Limited published data regarding the ROSA (Robotic Surgical Assistant) learning curve exist. This study evaluated the number of cases needed for an expert orthopaedic surgeon to master the ROSA system and match the operative time of robotic (raTKAs) and manual primary total knee arthroplasties (mTKAs).

**Methods:**

This retrospective comparative cohort study included two hundred patients with primary knee osteoarthritis. The study group consisted of an expert surgeon’s first 100 raTKAs. The control group included 100 patients that underwent mTKAs from the same surgeon during the same period. The consecutive cases in each group were divided into ten subgroups, each of 10 cases. The groups were comparable concerning age, sex, BMI and Kellgren–Lawrence classification. We compared each subgroup's operative time and complications in mTKA and raTKA groups. We performed a cumsum analysis to construct the ROSA learning curve.

**Results:**

The first non-significant difference between the mTKAs and raTKAs operative times was observed in the subgroup of 62 to 71 cases. Till then, the operative time has been significantly lower for the mTKA than the raTKA group. The following groups of tens analysis (8th, 9th and 10th) showed no operative time difference between groups. The learning curve analysis demonstrated that the surgeon switched to the mastering phase from case 73 onwards. The two groups had no complication rate differences.

**Conclusion:**

Our study demonstrated that about 70 cases are necessary for a senior surgeon to balance operative time between mTKAs and raTKAs using the ROSA system.

## Introduction

It has been reported that robotically assisted knee arthroplasty (raTKA) is more time-consuming, with a more extended learning curve than manual TKA (mTKA) [1]. The raTKA learning curve represents the necessary number of cases so that the surgeon achieves a consistent outcome or similar operative times with mTKA [2]. Two previous studies demonstrated a learning curve for the ROSA (RObotic Surgical Assistant) (Zimmer Biomet, Warsaw, IN, USA) system [2, 3]. In 2021, Vanlomen et al. estimated a learning curve of 6–11 cases, although, at this point, the ROSA operative time was notably higher than the mTKA. High volume (> 200 cases/year) orthopaedic surgeons that previously underwent ROSA cadaveric training performed the procedures [2]. In 2022, Bolam et al. calculated the ROSA learning curve of 5–15 cases. Fellowship-trained high-volume arthroplasty surgeons (> 100 TKA/year) that received four hours of ROSA theoretical training but had not performed any ROSA raTKA before the study were involved [3]. However, the mean surgical operative time was notably longer than in the previous study. We believe the above studies reported familiarization with the robotic system, which differs from time neutrality. Time neutrality is a rigorous definition meaning the same surgical time for either mTKA or raTKA technique.

Further investigation into raTKA is required. Our study aimed to estimate the learning curve of a fellowship-trained, high-volume, experienced orthopaedic surgeon to achieve comparable mean total operative time between the imageless ROSA raTKA and mTKA. We also compared complications during the surgeon's learning curve between raTKAs and mTKAs.

## Materials & methods

The Hospital Health Research Ethics Board approved this retrospective comparative study. All patients provided written informed consent before study inclusion.

### Patient population

Our study included two hundred posterior stabilized unilateral primary TKAs (NexGen Legacy, Zimmer Biomet, Warsaw, IN) performed by a high-volume arthroplasty surgeon. One hundred TKAs were the surgeon’s first consecutive raTKAs with the ROSA system (raTKA group) between January 2020 and January 2022. raTKAs were performed during the surgeon’s learning curve. During the same period, the first consecutive one hundred mTKAs performed by the same surgeon comprised the control group. The selection prerequisites for entering the study included (i) age > 18 years and (ii) patients experiencing symptomatic primary unilateral end-stage OA. Exclusion criteria consisted of (i) revision TKA, (ii) cases using another knee implant and (iii) patients < 18 years.

The manual or robotic technique was selected according to the patients’ preferences. The raTKA and mTKA advantages and disadvantages were well explained to the patients preoperatively, and the patient had the right to choose what the patient deemed the best freely.

### Surgeon robotic training

The surgeon underwent a Zimmer Biomet Institute Course, Cadaveric Practice Workshop: Rosa Knee with Persona Knee, that took place in Cologne, Germany, in August 2019. Before entering the study, he had never performed any ROSA raTKA nor gone to a mentor’s operating room.

### Operative time

The operative time did not involve the induction time to general anaesthesia; it was measured for both techniques from the first skin cut until the wound closure. The first cut for raTKAs was considered the trackers’ pinning. Before that and during the anaesthesia induction, the assisting surgeon set up and positioned the robotic device. After that, the chief surgeon pinned the trackers and performed the registration, surgical approach and all the other steps until skin closure. All necessary information was extracted from the intraoperative operation note file.

### Surgical technique

Fully cemented, posteriorly stabilized cobalt-chrome, metal-on-polyethene prostheses (NexGen Legacy, Zimmer Biomet, Warsaw, IN) with fixed-bearing were used for all cases. Pneumatic thigh tourniquet pressure of 300 mmHg was applied intraoperatively. The medial parapatellar approach was used in all cases. In robotic cases, the femoral (3.2 × 150 mm) and tibial (3.2 × 80 mm) pins have been positioned through different incisions than the main incision. The femoral pins were placed four fingers above and the tibial pins four fingers below the main knee incision. Our initial aim was to maintain a limb hip-knee-ankle angle of 180°, placing the components perpendicular to the mechanical femoral and tibial axis. The flexion and extension gaps were balanced using soft tissue releases, osteophytes removal and femoral ≤ 4 mm or tibial varus/valgus ≤ 2 mm cuts when needed. The final hip-knee-ankle angle ranged from neutral to 4° of varus. The operative technique for mTKA and raTKA has been previously described in detail [1].

### Surgical environment

We did not consider the surgical support staff learning curve (nurses, anaesthesiologist, etc.), which could impact the robotic method efficacy and intraoperative coordination. However, in every case, the same group of assisting surgeons, anaesthesiologists and nurses and the same industry representative were involved.

### Complications

All intraoperative and postoperative complications and specific robotic complications (periprosthetic tibial or femoral pin fractures, pin tract infection) and revisions were retrospectively recorded.

### Data collection and analysis

A specific procedure was followed to compare the mean intraoperative time between groups. Initially, the chronologically consecutive cases in each group were divided into ten subgroups, each of 10 cases. The first 10 cases comprised the first subgroup, the 11th to 20th cases, the second subgroup, etc. Then, the mean operative time of the relative subgroups in mTKA and raTKA groups was compared. We looked for the first pair of tens with a non-significant difference in the mean intraoperative time between raTKA and mTKA groups.

The “sliding window” technique was applied for a more detailed analysis [4]. The “window” moves one item at a time and constructs subarrays that overlay each other. Our analysis found the last important group and applied this method till the next group. Thus, ten new pairs of tens were created. On each of them, we performed statistical analysis to find the first non-significant one.

A cumsum (cumulative summation) analysis was also performed to achieve the visual representation of the ROSA learning curve. Cumsum values represent a running total of the differences between the value of each data point and a standardized target. Two Cumsum figures were generated, with standardized targets for the mean time of manual TKAs and the mean time of robotic TKAs, on the other hand.

IBM SPSS software (IBM, version 27.0) was used for the statistical analysis. Kolmogorov–Smirnov and Shapiro–Wilk tests were performed to determine the normality of the data distribution. Statistical tests were two-tailed. All *p *values less than 0.05 were considered statistically significant. Two-sided independent sample *t* test or Mann–Whitney *U*-test was utilized to compare normally and non-normally distributed continuous variables. Categorical variables were compared by the Chi-Square test (*x*^2^ test).

## Results

One hundred ROSA TKAs and 100 mTKAs were included in this study. Table 1 depicts the baseline characteristics of both groups’ patients. No significant difference in baseline characteristics, including age, sex, body mass index and Kellgren–Lawrence classification, was recorded between the groups or between the subgroups of tens (Table 1). The mean operative time of raTKAs (80.2 ± 14.2) min was significantly longer than the mean operative time of mTKAs (68.2 ± 8.6) min (Mann–Whitney test, *p* < 0.001).

**Table 1:** Comparative baseline characteristics between mTKA and raTKA groups

Characteristics		mTKA	raTKA	*p* value
Age*		73.6 (6.3)	74.4 (6.6)	0.343^@^
BMI*		27.4 (2.6)	28.2(3.1)	0.070^@^
Sex**	Men	34	39	0.462^#^
	Women	66	61	
Kellgren–Lawrence classification**	III	9	13	0.366^#^
	IV	91	87	

*mTKA* Manual total knee arthroplasty, *raTKA* Robotic total knee arthroplasty, *BMI* Body mass index

*The values are given as the mean with the standard deviation in parentheses

**The values are given as raw numbers

^@^Tests were performed using the Mann–Whitney test

^#^Tests were performed using *x*^2^ test

**Fig. 1:**
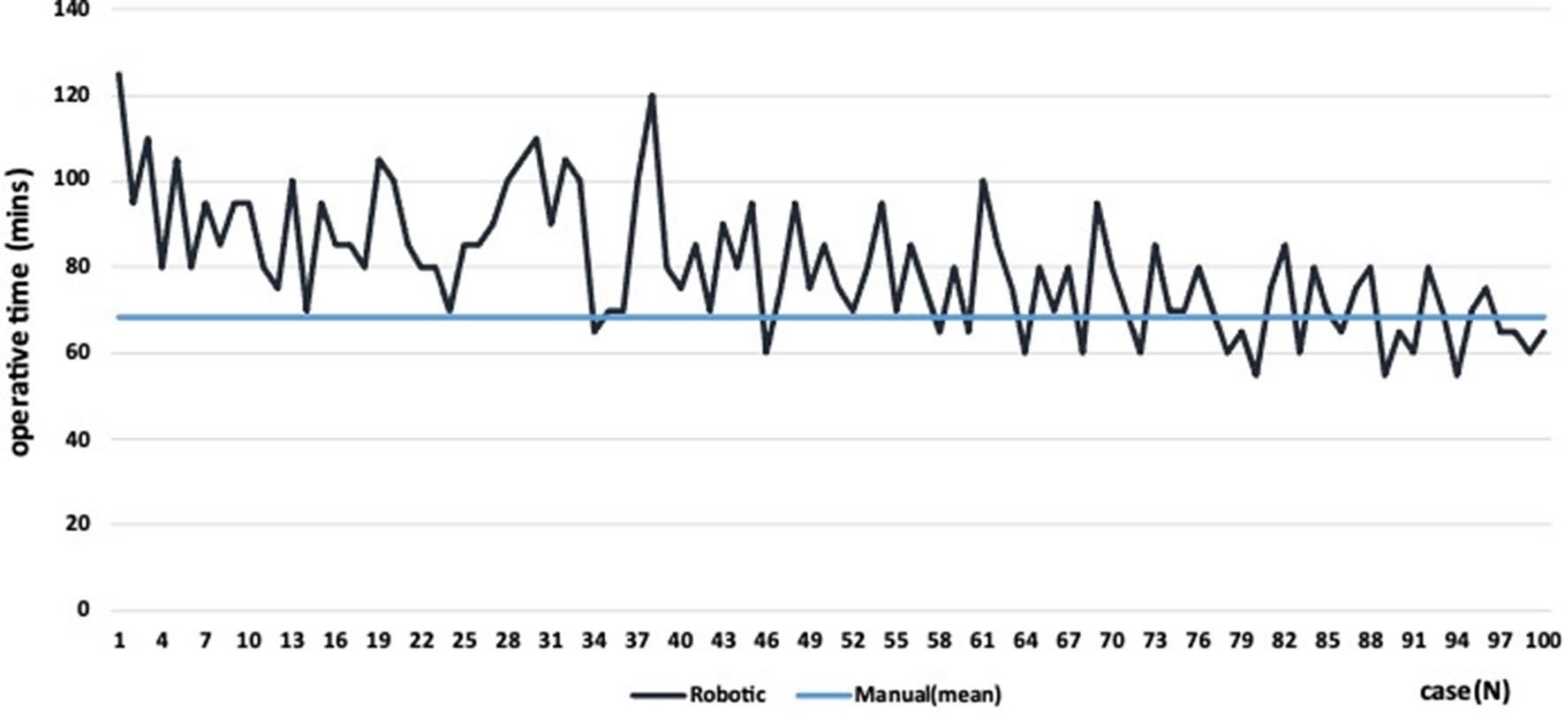
The operative time of all raTKAs (black line) in relation to the average operative time of mTKAs (blue line)

Table 2 shows multiple comparisons of ten patients' consecutive mTKA and raTKA subgroups. The mean operative time was statistically significantly lower for the mTKA than the raTKA subgroup till the seventh decade of patients between the groups (Table 2). Using the sliding window method, a non-significant difference was observed in the subgroups of 62 to 71 cases (*p* = 0. 063) between groups (Table 2). It is also worth noting that the mean operative time was significantly fewer for other subsequent subgroups (65–74 cases), favouring the mTKA group. However, we can certainly consider the seventh case decade as the range of equalization of the mean operative time of raTKA and mTKA groups. Figure 1 shows the operative time of all raTKAs to the mTKAs' average operative time, showing that the mTKAs' average line crosses the middle raTKAs function.

**Table 2:** Comparisons of the mean (median) operative time between consecutive groups of mTKA and raTKA groups (subgroups of tens)

Consecutive groups of cases	Operative time (mins)		*p* value^@^
	mTKA	raTKA	
	Mean	Mean	
1–10	68.50	96.50	< 0.001
11–20	72.00	87.50	0.011
21–30	70.50	89.00	0.001
31–40	70.50	87.50	0.029
41–50	66.50	81.00	0.004
51–60	65.50	76.00	0.023
61–70	66.50	78.50	0.035
Sliding window			
62–71	66.50	75.50	0.063
63–72	66.00	73.00	0.190
64–73	68.00	74.00	0.280
65–74	66.50	75.00	0.075
66–75	67.50	74.00	0.190
67–76	68.00	75.00	0.143
68–77	68.50	74.00	0.280
69–78	69.50	74.00	0.436
70–79	69.50	71.00	0.796
71–80	68.50	68.50	0.971
81–90	66.00	71.00	0.247
91–100	68.00	66.50	0.853

*mTKA* Manual total knee arthroplasty, *raTKA* Robotic total knee arthroplasty

^@^Tests were performed using the Mann–Whitney test

**Fig. 2:**
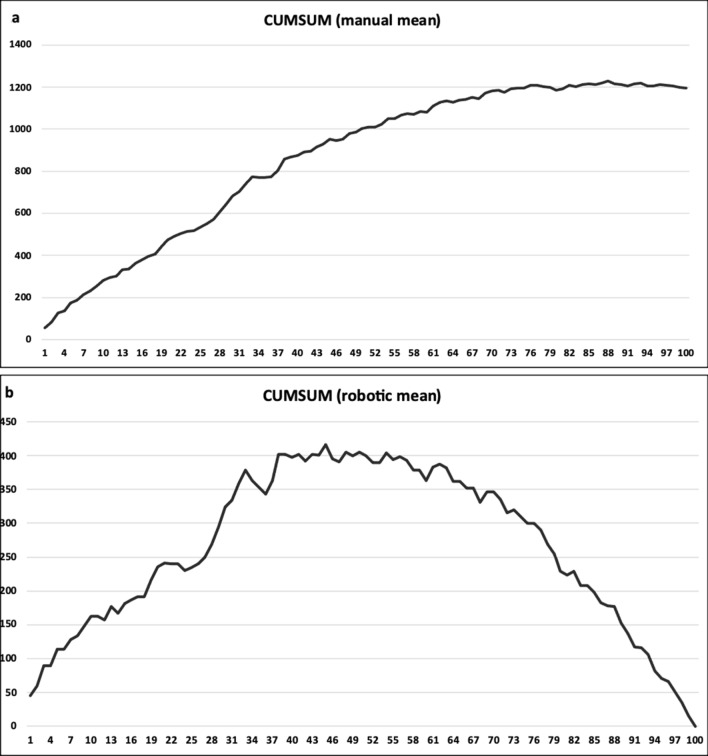
**a** and **b** Cumsum figures show the cumulative sum of the differences in the robotic operating times compared to a) the average operating time of manual TKAs (**a**) and (**b**) the average operating time of robotic TKAs (**b**)

Cumsum figures show the surgeon’s raTKAs learning curve and mainly the transition point from the learning to the mastering phase. In Fig. 2a, we have set the mTKAs average time as the standardized value, and we observe the progressive decrease of the function's slope from 65 to 70 and beyond. Figure 2b demonstrates the peak function at 43 cases, from which the curve decreases. At 75 cases, a sharp drop is seen, reflecting the raTKA learning curve refinement phase. Figure 2b can be divided into the following three phases: (a) learning phase I (case 1–case 43), (b) learning phase II (case 44–case 75), and (c) mastering phase (case 76–case100).

There were no complications related to the raTKA in this group of robotic cases. Two superficial infections were recorded, one per group.

## Discussion

Our main finding is that a fellowship-trained, high-volume, experienced orthopaedic surgeon equalized the mTKAs, and raTKAs mean operative time after seven decades of robotic cases. Our analysis also demonstrated that the senior surgeon switched to the mastering phase from the seventh decade of cases. This is reported for the first time in the literature. It is worth noting that there were no complication rate differences between the groups.

Two previous studies investigated the ROSA system learning curve. These studies evaluated the surgeons’ familiarization with the raTKA. Vanlomen et al. estimated a learning curve of 6–11 cases, with the times of robotic and manual surgeries remaining significantly different. The authors stated that the operative times are equalized later without further evidence [2]. Bolam et al. published a related survey, calculating the ROSA learning curve of 5–15 cases. The authors reported that the mean operative time of all raTKAs did not differ significantly from the mTKA group [3]. However, no table or graph was presented to confirm this fact, and it is worth noting that the mTKAs average time was much higher than the previous survey and ours. Our study demonstrated that 43 cases were necessary for the initial and 75 for the second learning curve phase of a fellowship-trained, high-volume orthopaedic surgeon with the ROSA system. Following the first 70 robotic cases, the senior surgeon entered the mastering phase, where the raTKAs and mTKAs mean operative time was balanced.

Several different steps between raTKAs and mTKAs may explain the operative time differences. In raTKAs, additional time is needed to set up the robotic units and the system registration and for the surgeons to familiarize themself with these steps and increase their confidence in the robotic system. In our study and practice, the ROSA setup is performed simultaneously during the patient introduction to anaesthesia and draping to diminish the extra time needed. On the other hand, the lack of multiple checks with alignment methods and cutting blocks, the reduced implant trailing time, and the more straightforward flow of surgical events may reduce the raTKA operative time. The question is whether the raTKA advantages outweigh the disadvantages and how this equation affects the average surgery time. Our study showed that although the mean initial raTKAs were significantly greater than the mTKAs time, the times are balanced during the surgeon’s mastering phase. The surgeon's confidence level improvement and mastering the technique explain the raTKAs gradual surgical time reduction. Thus, high volume experience with this new robotic method reduced the initial surgeon’s uncertainties and reinforced the philosophy of individualized alignment using the robot. This may not be the case for beginners or low-volume surgeons. Regular ROSA system use will improve the results even more.

The continuous improvement using raTKA has been confirmed by other robotic systems studies. Recent studies demonstrated that the learning curve of other raTKA systems was less than 25 cases [5–7]. In 2018, Sodhi et al. [8] reported that raTKA and mTKA operative times are equalized after about 40 cases. Another study demonstrated that robotic and manual surgery times were compensated after a 6-month learning curve and that the raTKAs operative times continued to decrease, resulting (after one year) in significantly shorter times than mTKAs [9]. Our ROSA system research showed an equalization of times and a trend of raTKAs operative time continuous improvement. The same surgical, anaesthesiologist, nursing team, or surgeon’s profile may have positively affected our results [10]. In our study, a senior surgeon has performed all TKAs, which must be considered for the outcomes’ interpretation. Besides, all surgeries were performed during the coronavirus disease 19 (COVID-19) pandemic lockdown period, which may have influenced the elective orthopaedic surgery rate in unpredictable ways.

No complication rate difference existed between the groups. No pin site complications were observed or other specific raTKA complications, which proves the procedure's safety. Increasing the surgical time has significant adverse implications for the patient, increasing the risk of periprosthetic infection and venous thromboembolism [11]. Besides, working with a robotic system, thus communicating constantly with a touch screen and a robotic arm, requires additional skills with high acquisition time, which is challenging to acquire [12]. The complications chances for raTKA mustn't increase, as this is the main criterion for integrating robotic technology into clinical practice. If proven that the new technology is entirely safe, the doctor's confidence and other parameters, such as the operative time, may improve.

This work has some limitations. Initially, the study's retrospective nature affects the data's quality. Still, as mentioned earlier, all surgeries were performed by a single physician and a specific medical team, limiting the ability to generalize the results safely. Also, all surgeries were performed during the COVID-19 lockdown period, which likely influenced elective surgeries and study results. Finally, regarding the expected selection bias, it should be noted that patients self-selected the surgery type based on their criteria. Contrary to the above, the study reliability increases by: (a) maintaining groups’ comparability and (b) preserving standard variables in each surgery (physician, technique, material).

In conclusion, raTKA with ROSA knee system semi-automates part of the surgical procedure but comes with a significant learning curve. An experienced orthopaedic surgeon needs about 70 surgeries to balance the robotic with conventional surgery time. Further analysis and research are to be done on this issue.
